# Social cohesion among healthcare workers during COVID-19: Qualitative research in Indonesia, Nepal, and Vietnam

**DOI:** 10.1016/j.ssmqr.2024.100404

**Published:** 2024-06

**Authors:** Ha Nguyen Thanh, Ida Ayu Sutrisni, Samita Rijal, Aakriti Pandey, Thao Phuong Tran, Ragil Dien, Yen Nguyen Thi Hong, Diana Timoria, Dewi Friska, Aria Kekalih, Claus Bogh, Abhilasha Karkey, Raph L. Hamers, Mary Chambers, Sonia Lewycka, Jennifer Ilo Van Nuil

**Affiliations:** a Oxford University Clinical Research Unit, Ho Chi Minh City, Viet Nam; b Oxford University Clinical Research Unit Indonesia, Faculty of Medicine Universitas Indonesia, Jakarta, Indonesia; c Oxford University Clinical Research Unit, Kathmandu, Nepal; d Oxford University Clinical Research Unit, Hanoi, Viet Nam; e Sumba Foundation, Sumba, Indonesia; f Department of Community Medicine, Faculty of Medicine Universitas Indonesia, Jakarta, Indonesia; g Centre for Tropical Medicine and Global Health, Nuffield Dept of Medicine, University of Oxford, Oxford, UK

**Keywords:** COVID-19, Healthcare workers, Qualitative, Social cohesion, Vietnam, Nepal, Indonesia

## Abstract

Existing literature has portrayed numerous challenges that healthcare workers (HCWs) faced during the COVID-19 pandemic, such as heightened risks of transmission against the scarcity of protective equipment, burgeoning workload, and emotional distress, to name a few. However, most studies explored HCWs' experiences at the individual level rather than examining the collective responses. Exploring these experiences could reveal the social-cultural locality of the pandemic while identifying the system constraints in public health emergencies. As part of a mixed-method study on COVID-19 pandemic impacts, we analysed qualitative interview data with 129 HCWs and health-related staff to explore their experiences during the pandemic between 2020 and 2021 in Vietnam, Indonesia, and Nepal. Using Bahers' sociological framework, Community of Fate, we describe five themes reflecting the formation of a community of HCWs and the social cohesion underlying their efforts to survive hardship. The first three themes characterise the HCW community of fate, including (1) Recognition of extreme work-related danger, (2) physical and figurative closures where HCWs restrict themselves from the outside world, (3) chronic ordeals with overwhelming workload and responsibilities, encompassing recurrent mental health challenges. Against such extreme hardship, cohesive bonding and social resilience are reflected through two additional themes: (4) a mutual sense of moral and professional duty to protect communities, (5) the vertical and horizontal convergence among HCWs across levels and among government departments. We discuss these HCWs’ challenges in relation to systemic vulnerabilities while advocating for increasing investment in public health and collaboration across government sectors to prepare for emergency situations.

## 1 Introduction

Healthcare workers (HCWs), including health-related staff, have been heavily impacted by the COVID-19 pandemic. A review of qualitative literature exploring frontline HCWs' experiences during COVID-19 and previous pandemics/epidemics identified eight main themes, including physical health, safety, security; workload; stigma; ethical/moral/professional dilemmas; personal and professional growth; support to and from others; knowledge and information; formal support (Billings et al., 2021). The authors argued that the experiences, framed mostly as challenges, identified from the 46 articles were similar over time (∼20 years), geographic contexts, and pathogens and focused mostly on individual level concerns, similar to literature surrounding HCWs’ experiences during pandemic times, for example, in the context of mental health (Ng et al., 2020; Spoorthy, 2020). Beyond the individual-level experiences, two concepts that tend to receive attention in the context of pandemics include social cohesion and community resilience, typically framed as having an influence on the ways in which pandemics play out in specific settings (Jewett et al., 2021; Ware, 2023). Further, exploring the impact of structural aspects on pandemic progress in a variety of settings could help to identify system vulnerabilities and the socio-cultural supports in settings where resources are constrained (Smith, 2020). All these insights, including a detailed exploration of social cohesion in particular, can inform future pandemic preparedness and strengthening of health system resilience (Connell, 2020; Jewett et al., 2021; Schillmeier, 2008).

In order to conceptualize social cohesion, we draw on Baehr's (2005) ‘communities of fate’ framework developed in response to the SARS outbreak in Hong Kong. Baehr argues that these communities will form when a variety of components are present during times of extreme stress (Baehr, 2005). According to the framework, outlined in Table 1 and described in section 3.0, the first four themes (i.e. danger recognition, moral density, trials, and closure) must be present for a community of fate to form, as well as resources, an axis of convergence, and related social rituals to instill social cohesion (Baehr, 2005). Existing studies showed that forms of social bonding among HCWs were an effective coping mechanism to overcome duress during the pandemic (Lee & Lee, 2020; Marey-Sarwan et al., 2021), but it was unclear how social cohesion was formed and experienced. Baehr (2005) only used the framework to describe the mutual experiences of Hong Kong people during SARS, not particularly of HCWs. During COVID-19, the framework was adapted by Montgomery and colleagues in response to the experiences of nurses working in four critical care units in London and suggested that resources as well as conditions for good care and staff well-being must be present for the ‘communities of fate’ to thrive (Montgomery et al., 2021). This suggestion may also be relevant to LMICs where health systems may be less developed.

**Table 1:** A brief description of the elements in the Community of Fate framework by Baehr (2005).

Danger Recognition	Perception of the evident danger that can create community suffering and threaten lives
**Moral density**	A feeling of social cohesion as their lives are connected to others, seeking common interests beyond individual and family's benefits
**Trial**	Chronic ordeals during the crisis, rather than acute shock
**Closure**	Physical and figurative restrictions on movement outside the boundaries of the communities, leading to “collective exile”
**Material and organisational resources**	Resources to combat crisis
**Axis of convergence**	A common language to talk about the crisis, social pride, and feeling of unity
**Social rituals**	Specific actions of the community formed during the crisis, separating them from routine life and the unaffected world

The objective of this analysis was to examine if and how communities of fate formed for HCWs working at various sites across three LMICs in South/Southeast Asia and to explore how different components of the framework helped the HCW communities thrive and provide the social cohesion required to survive, collectively, during the COVID-19 pandemic.

## 2 Material and methods

### 2.1 Study context

**Fig. 1:**
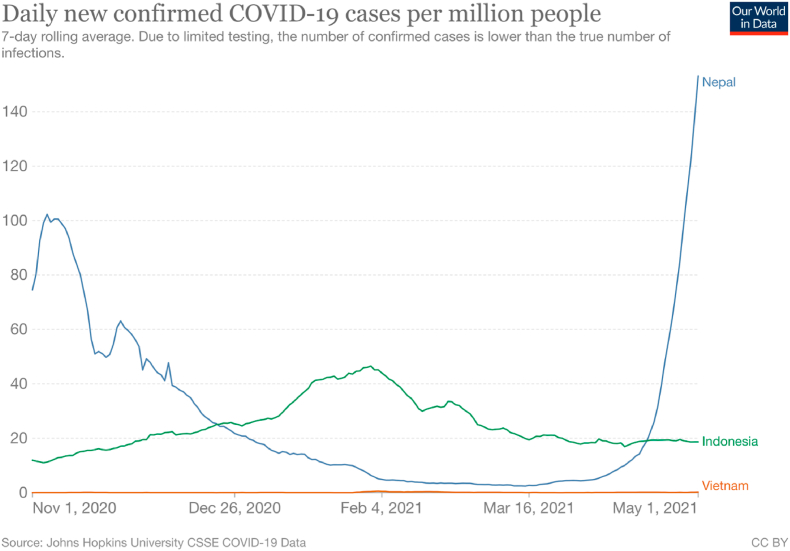
Cases per million for Indonesia, Nepal, and Vietnam during the SPEAR, phase 1 study period (November 2020 – April 30, 2021).

We used a subset of data collected as part of the ‘Social Science and Engagement Action Research’ (SPEAR) study, which was a social science study using quantitative, qualitative, and participant-led methods with HCW and community member participants over two phases across sites in Indonesia, Nepal, and Vietnam (Van Nuil et al., 2021). In Indonesia, we worked in three main provinces including, DKI Jakarta, West Java and Nusa Tenggara Timur. In Nepal, we conducted the study in four provinces including Bagmati, Gandaki, Lumbini, and Province 1. In Vietnam, our research spanned four provinces, including Ho Chi Minh city, Hanoi, Dak Lak, and Nam Dinh. Within each province, we selected a variety of healthcare contexts ranging from primary health centres to national infectious disease hospitals. The number of COVID-19 cases across the countries during the data collection period, November 2020-April 2021, are presented in Fig. 1 (Ritchie et al., 2020).

### 2.2 Sampling, data collection and analysis

We used purposive sampling to collect a range of experiences related to the study topics. With the help of our contacts within the study sites, we selected participants based on job role, work experience, department, gender, age groups and specific factors suggested by key informants at the study sites. We provided information about the study to leaders at the hospitals and clinics where the study took place and provided written and verbal information regarding the study prior to obtaining informed consent. All participants provided written or recorded verbal informed consent prior to participating in the study.

Experienced qualitative researchers working with the OUCRU team collected one in-depth interview with each participant, either in person or online, using a semi-structured interview guide developed by the investigators based on topics of relevance and literature of past epidemics. We started each interview with a narrative account of the participants’ experiences of COVID-19 and then followed up with questions driven by the study objectives and findings from subsequent interviews identified during weekly debrief sessions during data collection. We audio recorded, with explicit permission from participants, and transcribed the in-depth interviews into language spoken. During the transcription process, we removed identifying information. We translated the files into English and uploaded and managed them in NVivo12.

For all qualitative data collected the qualitative data team, including one to two researchers per country, first coded the files using a coding framework with both deductive and inductive codes (Dey, 1993; MacQueen & Guest, 2008; Miles et al., 2014). For the in-depth second cycle coding, a smaller group of researchers (HNT, IAS, SR, JVN) conducted the analysis. We started with the themes from the communities of fate framework (Baehr, 2005; Montgomery et al., 2021) by selecting the ‘relevant text’ from the first cycle coding (Auerbach & Silverstein, 2003) and then conducting multiple rounds of coding, discussions, and refinement of the original themes, first by country and then across the three countries. We used the overarching themes from the framework as the initial guide but then used open coding to divide the themes into context-specific sub-themes. During the analysis process we held weekly or fortnightly country meetings with the coding teams (HNT, IAS, SR) and the qualitative research lead (JVN). We also held multiple discussions with the researchers together as well as one meeting with the full SPEAR team for feedback and input into the preliminary findings.

## 3 Theory

According to Baehr, a community of fate will come into existence based on a situational, abrupt crisis rather than on its members' own decisions. This is particularly applicable to the times of COVID-19, and in this article, we show that the existing community of HCWs in Indonesia, Nepal, and Vietnam formed a community due to the emergence of the pandemic. We described the aspects of this community with three themes: *Danger recognition, Closure,* and *Ordeals,* which centered around the challenges and difficulties that HCWs encountered during responding to the pandemic. We then use the next two themes: *Moral Density* and *Axis of convergence,* to describe factors that helped HCWs cope with these negative extremities as a collective workforce. Reflecting throughout these five themes is a sense of social connectedness and mutual practices specific to the communities formed in times of crisis, a key element encompassed in Baehr's concept of community of fate termed “social rituals”. This annotated that the community of fate not only shares mutual distress but also take collective actions to tackle situations that arise. It is worth noting that another ingredient of the formation of the community of fate, *Material and organisational resources,* such as personal protection equipment (PPE) and human resources for COVID-19 intervention were a prominent concern in various contexts (Chemali et al., 2022; Koontalay et al., 2021). In our research, the issue surrounding resources for pandemic containment is also observed and contributes to experiences in different aspects of the HCW community of fate. Therefore, we discuss it as part of the five themes listed above instead of as a separate theme. Table 3 outlines the key differences between Baehr's (2005) and our themes and if and how the themes overlapped.

**Table 2:** Demographic data from in-depth interview participants, by country.

Gender	Indonesia	Nepal	Vietnam	Total
	N = 40 (%)	N = 43 (%)	N = 46 (%)	N = 129 (%)
Male	14 (35%)	17 (39.5)	17 (37%)	48 (37.2%)
Female	26 (65%)	26 (60.5%)	29 (63%)	81 (62.8%)
**Site**				
Urban	25 (62.5%)	23 (53.5%)	24 (52.2%)	72 (55.8%)
Rural	15 (37.5%)	20 (46.5%)	22 (47.8%)	57 (44.2%)
**Age group**				
20–29	7 (17.5%)	18 (41.9%)	5 (10.9%)	30 (23.3%)
30–39	16 (40%)	9 (20.9%)	11 (23.9%)	36 (27.9%)
40–49	5 (12.5%)	10 (23.3%)	7 (15.2%)	22 (17.1%)
≥50	3 (7.5%)	4 (9.3%)	6 (13%)	13 (10.1%)
Not stated	9 (22.5%)	2 (4.7%)	17 (37%)	28 (21.7%)
**Profession**				
Administration/Management (Hospital Director, Head of Primary Health, other management staff)	8 (20%)	2 (4.7%)	11 (23.9%)	21 (16.3%)
Doctor	6 (15%)	3 (7%)	6 (13%)	15 (11.6%)
Nurse/Midwife	12 (30%)	8 (18.6%)	16 (34.8%)	36 (27.9%)
Laboratory technician? staff?	3 (7.5%)	3 (7%)	1 (2.2%)	7 (5.4%)
Contact tracer/Health promotion officer/Health volunteers/Assistants	5 (12.5%)	5 (11.6%)	8 (17.4%)	18 (14%)
Cleaner/Driver/Security	2 (5%)	8 (18.6%)	3 (6.5%)	13 (10.1%)
Health Specialists (Nutritionist, Spiritual officer/Ayurveda medicine, Pharmacists)	4 (10%)	12 (27.9%)	1 (2.2%)	17 (13.2%)
Unknown positions	0 (0%)	2 (4.7%)	0 (0%)	2 (1.6%)
**Type of Health Facility**				
Hospital	7 (17.5%)	16 (37.2%)	23 50%)	46 (35.7%)
Primary health center	33 (82.5%)	20 (46.5%)	17 (37%)	70 (54.3%)
Others^a^	0 (0%)	7 (16.4%)	6 (13%)	13 (10.1%)

a Provincial Centre for Disease Control, Provincial Department of Health.

**Table 3:** Adapted Communities of Fate framework definitions, with key differences and overlap.

Baehr (2005) themes	SPEAR adapted sub-themes	Key differences and *thematic overlaps*
Danger recognition	• Transmission fears • Discrimination towards HCWs and families • Experiences in testing positive for COVID-19	HCWs were perceived as a danger by the community for their occupation. *Conflicting sentiments: Fear of HCWs from community/other staff/etc. but also the mantra of HCWs as heroes in Social rituals*
Closure-unable to escape from their predicament	• Isolation of HCWs in designated facilities • Separation of non-isolated HCWs from social networks, including reduction or absence of social rituals • Protection practice against COVID-19 infection and transmission (as a social ritual)	Does not have to be geographic closures. Emotional closure with a mindset to refrain themselves from social interaction. *HCWs isolated themselves as they internalised the stigma that they were a danger to the community.*
Trial – chronic ordeals	• Overload of work amount and responsibilities • Recurrent mental health issues	Focused on chronic impacts and continual difficulties, specific to HCWs *Psychological problems occurred as a result of different challenges described in all themes.*
Material and organizational resources	• Lack of protective equipment in the beginning • Creativity in the face of absent resources • COVID-19 volunteers	*Lack of resources increases danger recognition but also sets the stage for social cohesion (*e.g. *donations, locally created PPE).* *This theme was integrated into all other themes.*
Moral density - A sense of duty beyond the family unit	• Balancing safety and service duty, both individual and family • Pride and duty of being a HCW	Perceived professional duty as a healthcare worker motivated sacrifice. *Sense of duty often overrides fear and anxiety as brought out in ‘Danger recognition’ theme*
Axis of convergence -Common pride and unity	• Social language describing the pandemic as “war” and HCWs as “heroes”. • Horizontal convergence: mutual support among HCWs • Vertical convergence: donations from communities and joint support from other governmental sectors	*The rituals are engaged within and between communities, in this case*.

## 4 Results

### 4.1 Participant demographics

We enrolled 129 participants for in-depth interviews during the period of November 2020 until April 2021. Overall, 81 (62.8%) were female, over half of the participants were between the ages of 20–39 (66/128) and were working in a variety of HCW positions. About half of the participants (48.1%) were working in primary healthcare settings, 36.4% were working in hospitals, and the remainder (15.5%) were working in other health-related spaces. We included participants from both urban and rural settings (55.8% and 44.2%, respectively) to discern any differences between the two settings, as healthcare services in rural areas are usually resourced differently compared to urban areas, which may have led to different experiences for HCWs (Table 2). However, our data showed that the HCWs’ experiences between urban and rural sites had many similarities due to the extensive pandemic policies of the three governments that required responses at all levels in all local administration units of the public health network. In addition, when the cases peaked, we observed a similar inadequacy of resources in both rural and urban settings, which revealed the fragility of the whole health system in the three countries.

As we aim to describe the experiences of health workers on the frontline, we now refer to all participants as HCWs and the community described within this paper refers to the HCW community of fate, to distinguish from non-health frontline staff, e.g. volunteers or staff from other government sectors.

### 4.2 Danger recognition

For SARS in Hong Kong, Baehr (2005) refers to the general public's recognition of disease danger by their exposure to mass media about the outbreak and the national and international responses to containment. For HCWs during COVID-19, as they directly cared for infected patients or interacted with suspected cases, the risks of infection were even more conspicuous (Montgomery et al., 2021). In our research, the awareness of the HCW community toward COVID-19 danger was reflected through their heightened fear of perceived transmission risks, particularly due to lack of PPE, discriminatory attitudes towards them, and through their experiences of acquiring COVID-19.

Fear of transmission included HCWs’ worries about acquiring COVID-19 on the job. Fear was further extended by the lack of PPE in the early phases of the pandemic, but the necessity of needing PPE simultaneously caused more anxious feelings. PPE was both a symbol and risk of major infection and danger.

*Take wearing the PPE for example, it was not simply just putting them on, you had to carefully follow the safety procedure to wear it properly, and make sure you were safe from any risk of infection. This is why we were stressed by even the simplest tasks. We had to worry about if we did it properly, if it was safe, how to take it off to not let any of the aerosols from the suit on you, or how to avoid inhaling any of them. [Hospital Laboratory Technician, female, urban, Vietnam]*

Much has been investigated about the anxiety of frontline HCWs in caring for COVID-19 patients (Chemali et al., 2022), but in our research, we also observed the worries of less-represented health staff such as contact tracers, ambulance drivers, and community health workers.

*Overall, I was scared because although I was really careful for myself and my family, sometimes I was really worried if somehow I also got infected with COVID-19. I couldn’t know when I had got it. And because of my work, I also had to directly involve … like have contact with suspected cases … [Contact Tracer, male, rural, Vietnam]*

In rural settings, community health workers could have more concerns about transmission risks when their pandemic tasks entailed high exposure to their communities while they were not always provided with PPE. They often had to supply it by themselves.

*Participant: [Simultaneously] We go like we did before. [They] have distributed one mask anyway. We use our own sanitizer and go [to work].*

*Interviewer: Do they provide masks from here or do you have to buy yourself?*

*Participant: [Simultaneously] [We] are buying ourselves. [Community health worker, female, urban, Nepal]*

The risks of transmission were more apparent to the HCWs when they faced discrimination and stigma from communities. This included people avoiding them directly and blaming them for spreading COVID-19. HCWs were perceived as potential threats and a perceived danger.

*In the beginning, after COVID-19 started, even while going into the community, they [community] avoided me and discriminated. They behaved in ways, such as saying, ‘She works at the hospital, she has brought corona’. [Hospital Security Guard, male, urban, Nepal]*

*Some people look at us as if we carry some major diseases, they become so afraid to be near us. Yes, we feel pressure too, and stressed too, it’s like we are a disgrace. [Hospital Nurse, female, rural, Indonesia]*

In Vietnam, several participants said that HCWs were not allowed to go home due to the community's lack of understanding towards the risk of infection.

*There were times when it [stigma towards HCWs] was quite popular in the newspapers, and some of our HCWs were not even allowed to go back to their apartments as their landlords would not let them get back in the neighbourhoods. They were scared of people who were working in the hospital and afraid that they would get infected, they were just ignorant. [Hospital Ambulance Driver, male, urban, Vietnam]*

The participants also relayed their own stories of testing positive for COVID-19. The actual experiences of testing positive were common in interviews from both Nepal and Indonesia but not in Vietnam. At the time of data collection, the caseload in Vietnam was not as high, therefore fewer HCWs relayed these experiences. Here, the danger recognition centered on the fear of exposure and the implications of being someone who tested positive. In both Nepal and Indonesia, a component of danger recognition was experienced firsthand by those who had tested positive for COVID-19. The fear and danger relayed included fears about the burdens associated with acquiring COVID-19, the fear of death and leaving their families behind, and potentially the fear of their families experiencing stigma.

*I was slightly stressed because my report was negative a day before and the next day, [they] called me and said it was positive. [Clears throat] At that time, the majority [of people] didn’t [test] positive like now. So, I was kind of [worried] whether I was causing harm to others. [I: Okay] I also had some negative thoughts. Many of them responded positively, whereas many of them [said,] “I stayed with you. Or, I stayed at your side” like I transmitted it to them deliberately or, like they were afraid directly/indirectly.* [*Hospital Medical Officer, male, urban, Nepal]*

Further, in Indonesia, interviewees told us that their anxiety increased when they started to witness HCWs passing away.

### 4.3 Closure

A community of fate is bound by its members’ inability to escape, pushing them into a “collective exile”(Baehr, 2005). For SARS in Hong Kong, it was reflected in the fact that most people there were unable to leave Hong Kong during SARS due to international efforts to restrict travel. For COVID-19, it was a common practice for nations to close their borders, and similar decisions were made in Vietnam, Indonesia, and Nepal to restrict the expansion of the outbreaks. However, within each context, depending on the pandemic progress and infection clusters, communities at local levels were allowed to go outside, particularly during the phase of data collection. On the contrary, the experiences of HCWs were different, especially for those working in COVID-19 treatment facilities, quarantined centres or laboratories. They commonly stayed in institutions where they were working or in designated accommodations, not at home. For HCWs where they did not stay in separate accommodation, either because of the lack of facilities or no indicative policies, they still practised a certain amount of separation from their routine activities and social networks.

Participants, such as staff working in COVID-19 treatment hospitals or quarantine centres, often stayed in facilities, boarding houses, or hotels. Exile was physically bound for HCWs in the same isolated facilities, where they worked long shifts together, sometimes lived together, and other times stayed in isolation together.

*We were working night and day as there was no “going home” when you were already kept in isolation, and all of us were there for 14 days and could not go out. In addition to that, we had the daily task to look after the patients' conditions, taking their temperature twice a day for record. [CHC Staff, female, urban, Vietnam]*

They described a sense of separation from the outside world. They acknowledged the necessity of limiting interaction with people to protect their families and the public. For doctors treating COVID-19 patients, they still avoided neighbours and friends even though they had finished their quarantine period. The stigma about HCWs as a risky source of infection became internalised.

*I decided, I won't go back to my home frequently, because I'm afraid that I would transmit the virus to my home (family) … Finally, I lived in a boarding house. I stayed near the puskesmas (PHC), yeah because I'm worried about my family. Moreover, my parents are also … [old]. I feel gloomy. [CHC Staff, female, urban, Indonesia]*

Even when the isolated HCWs stayed in the same facility, they also felt the need to limit interactions with each other to prevent cross-infections. This created collective exile but also induced an individual closure for each member of the HCW community of fate.

*As for socializing, I can still interact with my colleagues, but we would have to wear our masks, and always keep our safe distance of at least two meters. [Hospital Paramedic, female, urban, Vietnam]*

The internalised stigma was also present for participants who did not stay inside designated accommodation. These were often HCWs in primary care settings, community health workers, or non-treatment staff such as contact tracers, or at some points in Indonesia, HCWs could choose to be isolated or not. They still made attempts to restrict themselves to their social circles. They stayed in separate rooms, ate at different hours, and avoided festivals, gatherings or celebrations to limit interactions. The transmission fear extended to bringing the infection home of their clothes, which often resulted in strict routines to avoid transmission, a sort of social ritual. They all shared a vigilance of caution in removing PPE, taking a shower, and washing their hands before interacting with outsiders. They showed a ready mentality to enter “exile” if they became close contacts of positive cases. In rural areas where primary health centres were responsible for public healthcare, staff were willing to be quarantined together if there were close contacts with infected cases.

*We said that if there was a positive case … We were prepared that if there was a positive case, we would bring our stuff in and stay, instead of going home [laughs]. [CHC Manager, female, rural, Vietnam]*

In this way, closure was not only physical confinement of the HCW community of fate but also a mutually psychological refrain from social connections, despite feeling lonely and missing their families.

### 4.4 Trial: chronic ordeals

When the pandemic began, substantial changes were compulsory in routine healthcare delivery to prioritise COVID-19 care and containment. There were various challenges that became persistent or sustained during their pandemic work. We focus on two prominent issues as they demanded attention for institutional support in all three countries: overload of work and mental health impacts.

#### 4.4.1 Overload of work

In all three countries, the health systems relied heavily on the labour of human resources. Pandemic responses were implemented by manual work through grassroots networks from the public health systems to the village level. For example, contact tracing was conducted manually by a team or in rural communes, COVID-19 communication was conducted by CHC staff and a network of community health workers. Also, participants generally reported the limitations in infrastructure that led to an overload of treatment, quarantine, and testing capacities across sites, especially during the peak of outbreaks. Thus, there was a surge in the responsibilities and continual additions of tasks assigned to HCWs to continually adapt to the pandemic progress.

*Workload increased due to the pipeline of COVID-19 patients, the referral systems, contact tracing work, providing health services in the health unit and community, coordination with other sectors, monitoring COVID patients with mild symptoms via telephone … [CHC Nurse, female, urban, Nepal]*

Also, whenever a colleague tested positive or was quarantined due to interacting with COVID-19 cases, the remaining team had to take over their tasks, which added burden on the overloaded staff, especially for areas with a limited number of HCWs.

*I’ve got five staff contracting COVID-19 together at once. We share the load, the PHC does not stop giving care. At the moment I have 15 staff suffering from COVID-19 [CHC Doctor, female, urban, Indonesia]*

Participants in Indonesia and Nepal also spoke about how there was a lack of time off and no leave mandate from the government.

*Some time ago, the health office issued a rule that at this time of peak conditions, no employees were on leave of work. So, when they are not on leave for work, how can they be refreshed? [Hospital Director, male, urban, Indonesia]*

In rural Vietnam, HCWs at primary settings such as the commune and village levels had to take part in the pandemic response and also deliver routine health programmes. A common narrative for these participants was of exhaustion due to the pressure from various stakeholders, the work that kept piling up, and the nonstop work because there were often COVID-19 associated tasks added to the routine work.

*I didn’t know if it was day or night, I didn’t know what a day off was. I didn’t have time for my family. Sometimes, I just came back home and ate a meal. Sometimes, I’m here [workplace] all day. I was making instant noodles at noon. Because there were just too many tasks. [Department of Health Staff, female, rural, Vietnam]*

In many situations, women complained about risks and challenges they faced during the pandemic work. The most frequently reported difficulty was HCW who were mothers, especially new mothers, having to stay separated from their children due to their occupational risks. Some women in Nepal complained about the frequent need to lift heavy equipment. In both Nepal and Vietnam, there were anxieties about safety to go out at night as women before/after late shifts.

#### 4.4.2 Mental health challenges

While we already described some aspects of mental health challenges previously, it is important to highlight as a specific subtheme because mental health issues were observed throughout all phases of the pandemic included in this research, although the specific issues in each phase may have been different. For example, when PPE supplies were inadequate, the extreme anxiety of transmission was prevalent while in the later phases with better PPE supply, the distress of wearing PPE for too long was observed. Other mental health issues that continually appeared throughout the pandemic included the stress of stigmatisation, loneliness in isolation, exhaustion from an overburdened workload but also uncertainty about the pandemic: *“Many people became depressed as they pondered what might happen next”* [*Medical Assistant, female, rural, Nepal*]. HCWs working directly with communities shared pressure to communicate with the public as not all people complied with precautionary measures.

Even for those who had experienced previous outbreaks, there were still heightened feelings about COVID-19 compared with the SARS outbreaks in 2003.

*Then when it came to actual work, the experience was really horrible, I was scared as I did not know a thing about it back then. Before this I had experience with SARS in 2003, I was also scared and worried but I did not feel as confused as I am now. [Hospital Paramedic, female, urban, Vietnam]*

This was not always the case, however, as four of the participants from an urban infectious diseases hospital in Vietnam had first-hand experience with H1N1, H5N1 and/or SARS previously, and their attitude was that pandemic control was part of their job.

*No one [from our department] was afraid [of the pandemic]. It’s like we were ready, we knew in advance, for example, if on the news it was said that the country had an outbreak, we were here [at the infectious disease hospital] getting ready. I don’t self-flatter but it was true. Because my department has been allocated the job of fighting pandemics. In the past at [the department], we also fought H5N1, SARS, yeah SARS. In general, we are all ready. [Hospital Nurse, female, urban, Vietnam 025]*

Despite pandemic control being part of their job, it didn't mean that they did not have to tend to their mental health, especially during (another) pandemic.

*Participant: H5N1 was in the 2000s, or 2010s, I couldn’t be sure. Was it H5N1, wasn’t it?*

*Interviewer: about 5 years ago?*

*Participant: 2012, I think …*

*Interviewer: 7 years?*

*Participant: probably so … I can’t remember*

*Interviewer: did your experience from the previous pandemic help you this time around?*

*Participant: In general …****I thought I had to protect my mental wellbeing first****… that’s right, the second thing is that we have to have sufficient knowledge.****We would be distracted if our mental health is not stable.****[HospitalDoctor, male, urban, Vietnam 022]*

Clearly there were varying impacts of pandemic uncertainty on frontline staff, but also for them, there were limited spaces to care for their mental well-being or they lacked institutional psychological support.

*It also got super busy and there was very little time to tend to emotional support for health staff as well as the patients, most of us only have time to support funding activities and logistics for daily meals and basic operations to ensure everyone feel more comfortable during that time, as the entire hospital was in isolation. [Hospital Social Worker, female, urban, Vietnam]*

*Because we had worked in a very difficult way at that time, I felt like the government should’ve given mental support at that time. [Hospital Lab Technician, female, urban, Nepal]*

Finally, there were stories about HCWs who decided to leave the job because of extreme distress so there is a limit.

*The wife of the village chief was enthusiastic initially, she was quite involved in COVID-19 handling but she eventually gave up due to frustrations and stresses. [CHC Nurse, female, urban, Indonesia]*

### 4.5 Moral density: balancing safety with HCW duties

Amidst the high risk of infection and the challenging working conditions, as mentioned in the three themes above, participants' narratives highlighted a mutual sacrifice to fulfill their duty of care as a healthcare worker. There was an overwhelming sense that the HCWs were doing their best to accommodate COVID-19 patients, as well as quarantined people, even though there was often a lack of resources and many difficulties. A participant from Vietnam spoke about the burdens of wearing PPE but in the end*, “It was all for the patients’ sake. You just have to take what comes with your job.”* [*Hospital Nurse, urban, Vietnam*]. Deliberately opting out of social gatherings was also a shared code of conduct among HCWs to reduce transmission risk as described in the theme “Closure”. Even when the social restrictions were eased, some HCW participants expressed a willingness to forgo their socialising needs for the prevention of COVID-19 transmission. For instance, an Indonesia HCW decided to postpone her wedding three times:

*My plan was to get married in February last year. In February, the early COVID-19 cases were really big, it was increasing. February continued to April, and to August. I postponed it three times. [CHC Staff, female, urban, Indonesia]*

Other HCWs in Vietnam and Nepal, HCWs also refrained from traditional social activities:

*I avoided visiting our neighbors on the Lunar New Year holiday. They might be worried as I worked for Mobile Action Team, so I just stayed at home. [Contact Tracer, male, rural, Vietnam]*

For participants, the motivation to sacrifice and overcome danger, fear, and anxiety stemmed from their perception of their duty as a healthcare professional. When asked about their perceived contributions to the pandemic response, a common sentiment was pride in their sacrifice to overcome multiple challenges to protect the communities.

*We have given 100% contribution to this hospital. We came to duty and did our duty. When everyone was at home, we did our duty. We cannot give a bigger contribution than this … We did our duty without considering our own lives. [Hospital Technician, female, urban, Nepal]*

*I feel proud about this [working as a healthcare worker]. This is because many of my friends had also left their jobs in the initial days due to fear of corona. We should understand our social responsibility and give continuity to this work. We have understood if not us, who will do it? We have been providing service to the patients. [HospitalMedical Officer, male, urban, Nepal]*

Others, including recent medical school graduates, volunteered to join the COVID-19 teams because they did not have a family at home or described how these experiences and opportunities would enhance their practical skills. These participants demonstrated that some sacrifice is acceptable and feeds into their passion of being an HCW.

*Nothing can scare me at all so when they first asked for volunteers to join the COVID team, I said I would do it, seeing as no one else wanted to … my kids are grown, it’s okay if I die. [Hospital Nurse, female, urban, Vietnam]*

For some participants, leaning on religion led to a sense of responsibility for the care of the community, despite being at high risk for COVID-19. This was more common in Nepal and Indonesia than in Vietnam. For example, one Indonesian nurse told us:

*I decided to help during COVID-19, considering from the side of my religion. Actually, I am afraid. When I want to join the army [the army was providing training for recruited HCWs who become volunteers in COVID-19 centers], I often wake up in the middle of the night, seeing my wife sleeping. Because I need to earn for living, and if I died, my wife will become a widow. It was in my mind too. […] But after reading some religious books, then finally I say .. we need faith to go further. [Hospital Nurse, female, urban, Indonesia]*

### 4.6 Axis of convergence

A community of fate shows a sense of unity. In this study, this is first reflected through shared language, i.e. how people refer to the crisis (Baehr, 2005). War terminology became a metaphor for COVID-19, similar to research conducted in other contexts (Manderson et al., 2021; Marcec et al., 2021; Marey-Sarwan et al., 2021) and in previous infectious outbreaks (Baehr, 2005; Marcec et al., 2021). In our study, controlling the pandemic was often referred to as “fighting the war”, or “the battle”, while HCWs were praised as being “heroes” or “warriors.” In this sense, a sentiment of civic unity emerged for all citizens to jointly combat the COVID-19. Axis of convergence was seen both horizontally among HCWs to support each other but also vertically, from other government departments and the public to help with the pandemic responses. Participants mentioned that mutual support from co-workers and management helped to manage the increased workload. Mutual support was linked to good teamwork, which helped to resolve complicated and emergent situations. This support happened at the level of the organization.

*But Alhamdulillah we have a team that has done our work to the maximum extent with the help of other colleagues, Alhamdulillah we passed each phase … Alhamdulillah, from the office, it is assisted by volunteers. [CHC Nurse, male, urban, Indonesia]*

But the support was also blended into the community.

*We can't work alone, of course, we can't work alone, so we empower all existing communities. For PHC representative, it's a cadre. We empower cadres, for … so we socialize the information of COVID-19 and vaccination to cadres. They are very helpful because we can't go directly to the field every day, right? [CHC Doctor, female, urban, Indonesia]*

Participants from Vietnam also spoke about how the community cooperated by following guidelines, helped to monitor and report ‘strangers’, and provided compliments and small gifts that lessened the HCWs hardships. The participants spoke about how these subtle acts from the community helped to tighten the bond of the communities of fate and perhaps extend it beyond the healthcare setting.

In all sites, there were reported donations and government resources that were pushed into the healthcare centres, hospitals, and into affected communities. There were various levels and types of donations depending on the timing and the specific context. For example, in Indonesia, due to increasing COVID-19 cases at the time of data collection, participants, particularly from urban sites, mentioned how the volunteer support provided by the government greatly helped the HCWs.

*At the beginning of the year, the cases were very high, even … the number of patients who had to wait for the treatment room in the emergency room was up to 30, even approaching 40 people. Until … the room had to be opened again for COVID-19. The doctors might be more mobile, but then we feel that we lack nurses. But thank God, because the DKI government has the policy to recruit volunteers, we are greatly helped by having volunteers. Volunteers, whether doctors, specialists, and also nurses, started to support us. [Hospital Mental Health Specialist, male, urban, Indonesia]*

During the data collection period, volunteers were not mentioned from Vietnamese or Nepali participants, but Vietnam HCWs expressed a strong sense of collaboration among different governmental bureaus to handle the pandemic at both national and local levels.

*Also a combination of the Committee, the health station and the community, and also police, women’s union staff, youth union staff, generally speaking all establishments and departments at the commune joined hands to fight the outbreak … Generally speaking one person couldn’t do it, but everybody could do it together. [CHC Staff, female, rural, Vietnam]*

The workforce participated in the response and formed a common identity as COVID-19 fighters. At lower levels, the COVID-19 containment measures were led by local governmental committees, indicating that the government placed distinctive importance on tackling the pandemic. Participants noted that this was the first time they witnessed so much effort and attention towards healthcare from different ministries in the government throughout all levels of management. Some participants also praised such unitedness as a key component to the internationally recognised successful response of Vietnam in 2020. Here their pride extended beyond the status of pandemic fighters but converged into the citizenship identity overcoming a national crisis.

*I was not only proud to be working in a reputable hospital, but I was proud to be a Vietnamese. [Hospital Social Worker, female, urban, Vietnam]*

## 5 Discussion

The use of the ‘Community of Fate’ framework helped us explore both the challenges and resilience of HCWs at collective levels in Vietnam, Nepal, and Indonesia during the COVID-19 pandemic from November 2020-April 2021. We found that the pandemic brought extreme fear, emotions, and physical danger, yet participants and their colleagues found support through social cohesion of a variety of forms. The metaphors and the ways in which participants discuss the COVID-19 pandemic demonstrated a sense of how these times were perceived both within and across the study contexts. In general, our study resonated with many existing studies about various difficulties that HCWs faced during the pandemic. This includes structural challenges such as lack of PPE and insufficient human and physical resources (Chemali et al., 2022; Hussain et al., 2021; Koontalay et al., 2021; Willis et al., 2021), as well as psychological issues such as fear and anxiety for personal risks of infection (Ozcan & Kirca, 2021; Sun et al., 2021), risks of transmitting COVID-19 to others (Koontalay et al., 2021), uncertainty about pandemic progress (Ozcan & Kirca, 2021), and pressure and exhaustion from increased workload (Jalili et al., 2021; Willis et al., 2021). Here we discuss two important narratives that our study contributes to the existing body of literature about preparedness for public health emergencies.

### 5.1 Health system preparedness

The pandemic revealed the fragility of the health system in the three countries where we conducted the study. The shortage of human resources led to increased workload and responsibility for HCWs in parallel to the limited infrastructure that recurrently challenged HCWs to adapt to pandemic progress. In addition, even though HCWs' mental distress was alarming, support for them remained limited. This could be explained by the general weakness in the provision of mental health services in the three countries (Cipta & Saputra, 2022; Nguyen et al., 2019; Rai et al., 2021), with such support becoming even more scarce in crisis times. Further, tending to HCWs' psychological problems is indispensable, even after pandemics ‘end’, as there was evidence of post-pandemic mental issues in HCWs (Hill et al., 2022). However, even if mental health support had been available, the overwhelming workload would likely have left HCWs with no space and time to utilise. In extreme situations, there were stories about HCWs at the study sites leaving their jobs, therefore there is a limit to the amount of ‘sacrifice’ one can manage for the wider community benefit. Given that the pandemic peaked more critically in the later phases than the data collection period in this research, the drain of healthcare workforce is likely more devastating. In fact, in Vietnam, media reported nearly 10,000 HCWs quit after the pandemic (Ministry of Health Bulletin, 2022). It, thus, posed a contentious question about the possibility of whether a HCW community of fate could reappear in a future public health crisis.

We also found challenges across different HCW groups that may be underreported in existing literature. For example, we were able to include narratives of health-related staff that were less represented in previous literature (Chemali et al., 2022), such as ambulance drivers, contact tracers, lab technicians or community workers. We also noted that these groups were often not provided with adequate protection measures, such as isolation facilities or PPE, even though they too had high-risk exposure. Similar findings were found in Smith's study in Indonesia (2020), that community midwives were not receiving PPE as they were not officially recognised HCWs. Female workers also expressed worries about occupational safety, in addition to the pressure for family caring placed in the cultural and social backgrounds of the three countries. Given that 70% of the global healthcare workforce is female, this issue needs institutional attention during public health crises (Mehta Sangeeta et al., 2021). More actions are necessary to protect HCWs against the physical risks of transmission. A better supply chain of PPE needs to be among the priorities in crisis preparedness as well as including frontline workers who were not officially recognised as ‘HCWs’ in previous outbreaks.

### 5.2 The need for collective solidarity

Against the extreme hardships, the frontline workers who were part of SPEAR study often found motivation and strength through their moral belief in their professions and the social cohesiveness among themselves. The pandemic workforce also lacked resources and infrastructure as in other settings, but they creatively managed by and for each other. However, we should not naturalise their sacrifice and efforts and overlook the impacts that the pandemic work induced on the HCWs' community of fate. One important issue is discrimination against frontline workers during the pandemic. The theme *Danger recognition* painted a contrasting picture that HCWs are not only “heroes only a distance” (Baehr, 2005) but also a “disgrace” to be near. Amidst receiving an outpour of praise and compliments from the communities and media, HCWs still faced discrimination and avoidance from their communities while they also felt the need to avoid social situations. While ostracism and stigma towards HCWs are also reported elsewhere (Koontalay et al., 2021), in our study, we observed that stigmatisation even spread to HCWs' families. Such discrimination not only affected HCWs’ motivations but also interfered with the pandemic-containing efforts; for example, in our contexts, HCWs had to go inside the communities to identify positive cases and close contacts. Discrimination towards HCWs in infectious epidemics are not unusual, as reported in studies about previous outbreaks of SARS, Ebola, MERS (Broom & Broom, 2017; Delamou et al., 2015; Salazar de Pablo et al., 2020; Verma et al., 2004). The recurrence of this problem throughout multiple epidemics reflects that it is still neglected in emergency preparedness. The health systems of the three countries could consider utilising digitalisation in surveilling and monitoring outbreaks, for example, digital contact tracing instead of manually tracing infected cases in the communities to minimise direct contact for frontline workers and potentially reduce stigma, however, digital contact tracing is not without issue. For example, a range of ethical issues have been identified in the literature including finding an appropriate balance between benefits and harms, issues related to privacy versus liberty, data ownership, how to account for cultural differences, and so on (Lucivero et al., 2020; Parker et al., 2020).

While a number of studies have shown that communities that show solidarity and collectivism during the pandemic had lower COVID-19 prevalence and mortality (Maaravi et al., 2021; Webster et al., 2021), some argued that solidarity can only take effect if it happens throughout society, and the public must support and respect frontline workers (Flynn, 2022). Our findings confirmed that cohesion only among HCWs would not be sufficient to help them cope with challenges and deal with the pandemic efficiently. In fact, when HCWs perceived solidarity on different axis, both across government sectors and between HCWs and the public, they felt more motivated, as reflected in the theme *axis of convergence*. In our settings, routine health services already existed at the grassroots level, therefore, HCWs and communities were already integrated to some extent. When we understand existing relationships and the ways in which social cohesion works, we can improve solidarity in times of uncertainty and crisis (Jewett et al., 2021).The stigma and discrimination described in this study was attributed mostly to a lack of understanding about viral transmission, not from the tension between HCWS and communities. Therefore, public communication and education is needed so that communities not only follow precautionary measures but also show their genuine respect to frontline workers to increase solidarity.

In addition, our research shows that support from government sectors and the communities was helpful in alleviating structural constraints during the pandemic. For example, collaboration among different governmental staff in frontline tasks such as contact tracing, surveillance, and provision of quarantine facilities reduced the physical and mental burden for HCWs. Volunteers recruited by the government were essential support during staff shortages in Indonesia. Such cohesion among different levels of the pandemic response teams is different to the findings of some existing research that highlighted a tension between frontline workers and managers where a ‘reality gap’ is drawn between policy and practical implementation (Willis et al., 2021). From the public side, communities also engaged in social rituals by sending gifts to support frontline staff. Donations were also contributed to poor communities to alleviate the burden on local authorities in basic need provisions for affected areas. In each country, the “community of fate” expanded outside the healthcare setting to include the wider public. Across sites, even though detailed experiences may vary culturally and differently according to the pandemic progress, similar patterns forming the community of fate among the three countries were observed in this study. In this way, the scope of community extended beyond borders and created an international web of communities. According to Baehr, social rituals separate communities from ‘normal’ life and the ‘world of the unaffected’, (Baehr, 2005), but for COVID-19, there wasn't a world of the unaffected.

### 5.3 Limitations

Using a framework can limit site-specific themes but the overarching framework helped us to compare across diverse sites, as well as compare with researchers who used the framework during COVID-19 in the UK (Montgomery et al., 2021) and during SARS in Hong Kong. The framework was a starting point for the development of themes and ideas, not the end point of discussion. As the framework focused on collective responses, we did not explore in-depth the heterogeneity in the experiences of different groups related to their ethnicity, urbanicity and work positions. As these characteristics may account for differences in challenges and vulnerabilities, we acknowledge that our findings may not portray a comprehensive picture of the pandemic responses in each country. Details on some of these topics and the experiences of HCWs in each country will be explored in more detail elsewhere. Nevertheless, in this paper we provide an overall view of shared collective experiences across three countries, particularly how social cohesion at a national level can mitigate challenges for HCWs that the pandemic brought about.

## 6 Conclusion

To prepare for any future pandemics, we strongly advocate for more investment in public health systems, particularly in LMICs like Vietnam, Nepal, and Indonesia, where the burden of tackling outbreaks often falls extensively on HCWs at primary care settings. In our study, overall, we found that HCWs formed a variety of communities of fate within and between contexts. These communities provided a (short-term?) mechanism for survival during an uncertain global pandemic. Whether or not these communities will result in longer-term communities has yet to be determined. However, the importance of the system-level consequences of the communities can provide models for future changes.

## Ethics approvals

The National Hospital for Tropical Diseases Ethics Committee (Hanoi, Vietnam), the Hospital for Tropical Diseases Ethics Committee (Ho Chi Minh City, Vietnam), the Ethics Committee of Nepal, Nepal Health Research Council (Kathmandu, Nepal), Patan Hospital Ethics Committee- Institutional Review Committee (Lalitpur, Nepal), Research Ethics Committee of the Faculty of Medicine, University of Indonesia (Jakarta, Indonesia) and Oxford Tropical Research Ethics Committee (Oxford, UK) all reviewed and approved SPEAR study.

## CRediT authorship contribution statement

**Ha Nguyen Thanh:** Data curation, Formal analysis, Writing – original draft. **Ida Ayu Sutrisni:** Data curation, Formal analysis, Writing – review & editing. **Samita Rijal:** Data curation, Formal analysis, Writing – review & editing. **Aakriti Pandey:** Data curation, Writing – review & editing. **Thao Phuong Tran:** Formal analysis, Writing – review & editing. **Ragil Dien:** Data curation, Project administration, Writing – review & editing. **Yen Nguyen Thi Hong:** Data curation, Formal analysis, Project administration, Writing – review & editing. **Diana Timoria:** Data curation, Writing – review & editing. **Dewi Friska:** Conceptualization, Supervision, Writing – review & editing. **Aria Kekalih:** Conceptualization, Supervision, Writing – review & editing. **Claus Bogh:** Supervision, Writing – review & editing. **Abhilasha Karkey:** Conceptualization, Funding acquisition, Supervision, Writing – review & editing. **Raph L. Hamers:** Conceptualization, Funding acquisition, Supervision, Writing – review & editing. **Mary Chambers:** Conceptualization, Funding acquisition, Supervision, Writing – review & editing. **Sonia Lewycka:** Conceptualization, Methodology, Supervision, Writing – review & editing, Funding acquisition. **Jennifer Ilo Van Nuil:** Conceptualization, Formal analysis, Funding acquisition, Methodology, Supervision, Writing – original draft, Writing – review & editing.

## Declaration of competing interest

The authors declare that there are no competing interests.

## References

[bib1] Auerbach C.F., Silverstein L.B. (2003).

[bib2] Baehr P. (2005). Social extremity, communities of fate, and the sociology of SARS. Archives Européennes de Sociologie.

[bib3] Billings J., Ching B.C.F., Gkofa V., Greene T., Bloomfield M. (2021). Experiences of frontline healthcare workers and their views about support during COVID-19 and previous pandemics: A systematic review and qualitative meta-synthesis. BMC Health Services Research.

[bib4] Broom A., Broom J. (2017). Fear, duty and the moralities of care: The ebola 2014 threat. Journal of Sociology.

[bib5] Chemali S., Mari-Sáez A., El Bcheraoui C., Weishaar H. (2022). Health care workers' experiences during the COVID-19 pandemic: A scoping review. Human Resources for Health.

[bib6] Cipta D.A., Saputra A. (2022). Changing Landscape of mental health from early career Psychiatrists' Perspective in Indonesia. Journal of Global Health Neurology and Psychiatry.

[bib7] Connell R. (2020). COVID-19/Sociology. Journal of Sociology.

[bib8] Delamou A., Beavogui A.H., Kondé M.K., Van Griensven J., De Brouwere V. (2015).

[bib9] Dey I. (1993).

[bib10] Flynn A.V. (2022). Solidarity and collectivism in the context of COVID-19. Nursing Ethics.

[bib11] Hill J.E., Harris C., Danielle L.C., Boland P., Doherty A.J., Benedetto V., Gita B.E., Clegg A.J. (2022).

[bib12] Hussain M., Begum T., Batul S.A., Tui N.N., Islam M.N., Hussain B. (2021). Healthcare workers during the COVID-19 pandemic: Experiences of doctors and nurses in Bangladesh. The International Journal of Health Planning and Management.

[bib13] Jalili M., Niroomand M., Hadavand F., Zeinali K., Fotouhi A. (2021). Burnout among healthcare professionals during COVID-19 pandemic: A cross-sectional study. International Archives of Occupational and Environmental Health.

[bib14] Jewett R.L., Mah S.M., Howell N., Larsen M.M. (2021). Social cohesion and community resilience during COVID-19 and pandemics: A Rapid scoping review to inform the united nations research Roadmap for COVID-19 Recovery. International Journal of Health Services.

[bib15] Koontalay A., Suksatan W., Prabsangob K., Sadang J.M. (2021).

[bib16] Lee N., Lee H.J. (2020). South Korean nurses' experiences with patient care at a covid-19-designated hospital: Growth after the frontline battle against an infectious disease pandemic. International Journal of Environmental Research and Public Health.

[bib17] Lucivero F., Hallowell N., Johnson S., Prainsack B., Samuel G., Sharon T. (2020). COVID-19 and contact tracing apps: Ethical challenges for a social Experiment on a global Scale. Journal of bioethical inquiry.

[bib18] Maaravi Y., Levy A., Gur T., Confino D., Segal S. (2021). “The Tragedy of the commons”: How individualism and collectivism affected the spread of the COVID-19 pandemic. Frontiers in Public Health.

[bib19] MacQueen K., Guest G., MacQueen K., Guest G. (2008). Handbook for team-based qualitative research.

[bib20] Manderson L., Burke N., Wahlberg A. (2021).

[bib21] Marcec R., Majta M., Likic R. (2021). Will vaccination refusal prolong the war on SARS-CoV-2?. Postgraduate Medical Journal.

[bib22] Marey‐Sarwan I., Hamama‐Raz Y., Asadi A., Nakad B., Hamama L. (2021). “It's like we’re at war”: Nurses' resilience and coping strategies during the COVID‐19 pandemic. Nursing Inquiry.

[bib23] Mehta S., Machado F., Arthur K., Laurent P., Marc M., Élie A., Margaret H. (2021). COVID-19: A heavy toll on health-care workers. The Lancet Respiratory Medicine.

[bib24] Miles M., Huberman A., Saldana J. (2014).

[bib25] Ministry of Health Bulletin (2022). https://moh.gov.vn/tin-tong-hop/-/asset_publisher/k206Q9qkZOqn/content/18-thang-co-9-680-nhan-vien-y-te-xin-thoi-viec-bo-viec-bo-y-te-chi-ra-4-nguyen-nhan-chinh.

[bib26] Montgomery C.M., Humphreys S., McCulloch C., Docherty A.B., Sturdy S., Pattison N. (2021). Critical care work during COVID-19: A qualitative study of staff experiences in the UK. BMJ Open.

[bib27] Ng Q.X., De Deyn M.L.Z.Q., Lim D.Y., Chan H.W., Yeo W.S. (2020).

[bib28] Nguyen T., Tran T., Tran H., Tran T., Fisher J. (2019). Innovations in global mental health.

[bib29] Ozcan S., Kirca N. (2021). Feelings, thoughts, and experiences of healthcare professionals who recovered after being diagnosed with COVID-19, a phenomonological study.

[bib30] Parker M.J., Fraser C., Abeler-Dörner L., Bonsall D. (2020). Ethics of instantaneous contact tracing using mobile phone apps in the control of the COVID-19 pandemic. Journal of Medical Ethics.

[bib31] Rai Y., Gurung D., Gautam K. (2021). Insight and challenges: Mental health services in Nepal. BJPsych International.

[bib33] Ritchie H., Mathieu E., Rodes-Guirao L., Appel C., Giattino C., Ortiz-Ospina E., Hassell J., Macdonald B., Beltekian D., Roser M. (2020).

[bib34] Salazar de Pablo G., Vaquerizo-Serrano J., Catalan A., Arango C., Moreno C., Ferre F., Shin J.I., Sullivan S., Brondino N., Solmi M., Fusar-Poli P. (2020).

[bib35] Schillmeier M. (2008). Globalizing risks - the cosmo-politics of SARS and its impact on globalizing sociology. Mobilities.

[bib36] Smith C. (2020). The structural vulnerability of healthcare workers during COVID-19: Observations on the social context of risk and the equitable distribution of resources. Social Science & Medicine.

[bib37] Spoorthy M.S. (2020).

[bib38] Sun P., Wang M., Song T., Wu Y., Luo J., Chen L., Yan L. (2021).

[bib43] Van Nuil J.I., Friska D., Kekalih A., Bhandari A.R., Bogh C., Brindle H. (2021). COVID-19 Social Science and Public Engagement Action Research in Vietnam, Indonesia and Nepal (SPEAR): Protocol for a mixed methods study exploring the experiences and impacts of COVID-19 for healthcare workers and vulnerable communities. Wellcome Open Research.

[bib39] Verma S., Chan Y.H., Deslypere J. (2004). Article in annals of the academy of medicine Singapore.

[bib40] Ware P. (2023). Social cohesion and Covid-19: An integrative review. medRxiv.

[bib41] Webster G.D., Howell J.L., Losee J.E., Mahar E.A., Wongsomboon V. (2021). Culture, COVID-19, and collectivism: A paradox of American exceptionalism?. Personality and Individual Differences.

[bib42] Willis K., Ezer P., Lewis S., Bismark M., Smallwood N. (2021). “Covid just amplified the cracks of the system”: Working as a frontline health worker during the COVID-19 pandemic. International Journal of Environmental Research and Public Health.

